# Optimization of bacterioruberin production from *Halorubrum ruber* and assessment of its antioxidant potential

**DOI:** 10.1186/s12934-023-02274-0

**Published:** 2024-01-03

**Authors:** Chi Young Hwang, Eui-Sang Cho, Sungjun Kim, Kyobum Kim, Myung-Ji Seo

**Affiliations:** 1 Department of Bioengineering and Nano-Bioengineering, Graduate School of Incheon National University, Incheon, 22012 Republic of Korea; 2 Department of Chemical and Biochemical Engineering, Dongguk University, Seoul, 04620 Republic of Korea; 3 Division of Bioengineering, Incheon National University, Incheon, 22012 Republic of Korea; 4 Research Center for Bio Materials & Process Development, Incheon, 22012 Republic of Korea; 5 MJ BIOLAB, Inc, Incheon, 21999 Republic of Korea

**Keywords:** Bacterioruberin, *Halorubrum ruber*, Carotenoid, Optimization, Antioxidant

## Abstract

Haloarchaea produce bacterioruberin, a major C_50_ carotenoid with antioxidant properties that allow for its potential application in the food, cosmetic, and pharmaceutical industries. This study aimed to optimize culture conditions for total carotenoid, predominantly comprising bacterioruberin, production using *Halorubrum ruber* MBLA0099. A one-factor-at-a-time and statistically-based experimental design were applied to optimize the culture conditions. Culture in the optimized medium caused an increase in total carotenoid production from 0.496 to 1.966 mg L^− 1^ Maximal carotenoid productivity was achieved in a 7-L laboratory-scale fermentation and represented a 6.05-fold increase (0.492 mg L^–1^ d^–1^). The carotenoid extracts from strain MBLA0099 exhibited a 1.8–10.3-fold higher antioxidant activity in vitro, and allowed for a higher survival rate of *Caenorhabditis elegans* under oxidative stress conditions. These results demonstrated that *Hrr. ruber* MBLA0099 has significant potential as a haloarchaon for the commercial production of bacterioruberin.

**Supplementary Information:**

The online version contains supplementary material available at 10.1186/s12934-023-02274-0.

## Introduction

Carotenoids are isoprenoids derived from the terpenoid biosynthetic pathway that play various roles in nature [1]. These compounds are generally produced by plants, algae, bacteria, archaea, fungi, and yeast *via* biosynthetic pathways. In photosynthetic organisms, carotenoids function as antioxidants that protect the photosynthetic machinery from damage induced by free oxygen radicals [2, 3]. In microorganisms, carotenoids enhance the mechanical strength and flexibility of the cell membranes, protect against lipid peroxidation, and maintain cell viability in extreme environments [4–6].

Haloarchaea are a unique group of microorganisms that are adapted to survive under high-salt conditions such as in saline lakes, salterns, seawater, and salted fermented food [7]. Because of their rigid cell membranes by compatible solutes and the salt-in strategy, they can survive in environments with high-salt concentrations. Therefore, halophilic archaea are free from break-in osmotic lysis caused by water moving in a high-salt environment [8, 9]. Carotenoids in haloarchaeal cellular membranes may also support cellular adaptation to hypersaline environments by functioning as water barriers and allowing ions and oxygen molecules to move into the cell membrane [10].

Carotenoid production from microorganisms offers an attractive alternative to plant-based carotenoids because of its shorter life cycle, the possibility of round-the-year production, the possibility of producing novel carotenoids, and the ease of maintaining controlled conditions during fermentation [11]. In addition, waste substrates can be converted into valuable carotenoids, thus making waste an asset. Carotenoid production can be increased by modulating various environmental parameters such as pH, temperature, carbon and nitrogen sources, and salinity, employing culture optimization [12, 13].

Bacterioruberin (BR) is a major C_50_ carotenoid produced by haloarchaea that has potential applications in the food, cosmetic, and pharmaceutical industries according to its antioxidant properties [14]. The ability of carotenoids to rapidly generate triplet-state carotenoids while inactivating the active oxygen species was outstanding. It is widely acknowledged that an increasing number of conjugated double bonds corresponds to a larger quenching activity for singlet oxygen [15]. Therefore, BR is a better singlet oxygen quencher than the C_40_ carotenoids generated by most photosynthetic organisms, because of its high number of conjugated double bonds.

Thus, this study aimed to optimize the conditions for total carotenoid production with outstanding antioxidant effects. By culturing *Halorubrum ruber* MBLA0099, we employed one-factor-at-a-time (OFAT) and statistical response surface methodology (RSM) to study the effects of various environmental parameters, including carbon and nitrogen sources, carbon/nitrogen (C/N) ratio, pH, inoculum size, and incubation time, on carotenoid production. The optimized culture conditions were further investigated in a laboratory-scale fermentation, to maximize carotenoid productivity. Finally, the in vitro and in vivo antioxidant properties of the carotenoid extract produced by strain MBLA0099 (BR extract) were evaluated.

## Materials and methods

### Strain and flask cultivation

*Hrr. ruber* MBLA0099 was isolated from a seawater sample collected from the Yellow Sea in Sorae, Incheon, Republic of Korea [16]. The strain was stored at -80 °C in 25% (w/v) glycerol stock solution. The strain was deposited in Korean Collection for Type Cultures (KCTC) and Japan Collection of Microorganisms (JCM) with accession numbers KCTC 4296 and JCM 34,701, respectively. The strain was cultured in ATCC 1176 medium containing 156 g L^–1^ NaCl (Duksan, Seoul, South Korea), 5.0 g L^–1^ yeast extract (BD Difco, Detroit, MI, USA), 1.0 g L^–1^ glucose (Samchun, Pyeongtaek, South Korea), 13 g L^–1^ MgCl_2_·6H_2_O (Daejung, Siheung, South Korea), 20 g L^–1^ MgSO_4_·7H_2_O (Daejung, Siheung, South Korea), 4.0 g L^–1^ KCl (Daejung, Siheung, South Korea), 0.5 g L^–1^ NaBr (TCI chemicals, Tokyo, Japan), 0.2 g L^–1^ NaHCO_3_ (Sigma-Aldrich, St. Louis, MO, USA), 1.0 g L^–1^ CaCl_2_·6H_2_O (Sigma-Aldrich, St. Louis, MO, USA), and adjusted to pH 7.0 using a 1 M Tris-based buffer solution (Sigma-Aldrich, St. Louis, MO, USA). The seed culture was prepared in 20 mL of liquid medium in a 50-mL conical tube and incubated until 2 d of growth was achieved. This culture was then used as the inoculum [at a concentration of 1% (v/v)] for the main culture. The cultures were cultivated under standard aerobic conditions in a 200-mL Erlenmeyer flask (working volume, 80 mL), with rotation at 200 rpm, 37 °C, for 6 d to reach peak carotenoid production. All of flask cultures were performed using SW-250B2 shaking incubator (Gaon Science, Bucheon, South Korea).

### Screening of medium components by using one-factor-at-a-time

The conventional OFAT optimization approach was used to evaluate the effects of optimal nitrogen and carbon sources, initial carbon levels, and C/N ratio on total carotenoid production. The yeast extract was replaced with organic [beef extract (KisanBio, Seoul, South Korea), peptone (KisanBio, Seoul, South Korea), fish peptone (HiMedia, Mumbai, India), tryptone (Duchefa, Haarlem, Netherlands), and casamino acid (BD Difco, Detroit, MI, USA) and inorganic [monosodium glutamate (Sigma-Aldrich, St. Louis, MO, USA), ammonium sulfate (Sigma-Aldrich, St. Louis, MO, USA), ammonium citrate (Sigma-Aldrich, St. Louis, MO, USA), and sodium nitrate (Sigma-Aldrich, St. Louis, MO, USA)) nitrogen sources (at a concentration of 5 g L^–1^) in the culture medium, as the sole nitrogen source. Similarly, glucose was replaced with fructose (Duksan, Seoul, South Korea), galactose (Samchun, Pyeongtaek, South Korea), glycerol (LPS Solution, Seoul, South Korea), lactose (Duksan, Seoul, South Korea), maltose (Junsei Tokyo, Japan), mannose (Samchun, Pyeongtaek, South Korea), mannitol (Samchun, Pyeongtaek, South Korea), sucrose (Samchun, Pyeongtaek, South Korea), or sodium acetate (Sigma-Aldrich, St. Louis, MO, USA) (at a concentration of 1 g L^–1^) in the culture medium, as the sole carbon source. To optimize the effect of the initial carbon source, concentrations of 0, 1, 2, 5, 10, 20, 40, and 80 g L^–1^ were tested. To identify the optimal C/N ratio, carbon and nitrogen sources were used at ratios of 10:1, 5:1, 3:1, 2:1, 1·5:1, 1:1, 1:1·5, 1:2, 1:3, 1:5, and 1:10, respectively. The effect of NaCl concentration was monitored in the range of 7.5–30% (w/v), at intervals of 2.5%. For proper magnesium ion supplementation, only MgCl_2_·6H_2_O (30 g L^–1^) and only MgSO_4_·7H_2_O (35 g L^–1^) were added to the modified ATCC 1176 media without magnesium supplementation. Supplementation amounts were added based on the molecular weights of MgCl_2_·6H_2_O and MgSO_4_·7H_2_O, according to the ratios of magnesium ions calculated equally compared to the basal ATCC 1176 medium. Finally, the best medium constitution obtained using the OFAT methodology was named ATCC 1176O medium.

### Optimization of culture conditions using a statistical approach

To identify the significant factors affecting total carotenoid production, different medium components were assessed using the Plackett–Burman experimental design (PBD) [17, 18]. Additionally, inoculum size and incubation time were selected as experimental factors, which are essential for achieving optimal production and influencing the rate of the fermentation process. The central level (0) was designated as the midpoint based on the ATCC 1176O medium. All medium components and physical factors were investigated at low (–1) and high (+ 1) levels (Table 1). Each independent factor was successfully screened in 12 experimental trials, plus 3 central points. Consequently, 15 runs were designed, and the PBD for this experiment was designed and analyzed using the Minitab statistical software version 18·1 (Minitab, State College, PA, USA). The PBD that followed the first-order model was calculated using Eq. (1):

**Table 1:** The level of factors, their codes, and actual values involved in plackett-burman experiment design

Code	Factor (units)	Low (-1)	Central (0)	High (+ 1)
**X**_**1**_	Sucrose (g L^− 1^)	0.5	2.0	3.5
**X**_**2**_	Yeast extract (g L^− 1^)	3.86	7.73	11.25
**X**_**3**_	MgSO4·7H_2_O (g L^− 1^)	10	35	60
**X**_**4**_	pH	6.0	7.0	8.0
**X**_**5**_	Incubation time (days)	3	6	9
**X**_**6**_	Inoculum volume (%)	1	5.5	10
**X**_**7**_	KCl (g L^− 1^)	2.0	4.0	6.0
**X**_**8**_	CaCl_2_·6H_2_O (g L^− 1^)	0.5	1	1.5
**X**_**9**_	NaCl (g L^− 1^)	125	200	275

1 $$Y = {\beta _0} + \sum\limits_{i = 1}^k {{\beta _i}} {X_i}$$

Where Y is the response of the dependent variable (carotenoid production), X_i_ is the independent variable, β_i_ is the linear coefficient, and β_0_ is the intercept of the model.

After the dominant factors were identified using PBD, RSM was applied to optimize the screened variables for enhanced carotenoid production by applying the Central Composite Design (CCD). Consequently, a total of 5 factors were selected from PBD – pH, yeast extract (g L^–1^), NaCl (g L^–1^), inoculum volume (%), and incubation time (days), for further optimization of carotenoid production. Owing to a full factorial CCD of 2^5^=32, 6 central points, and (2 × 5 = 10) star points, 48 experimental runs were designed. In this design, the star points are at the center of each face of the factorial space. Three different design levels were implemented to assess each factor, including a combination of factorial points (–1, + 1) and a central point (0) (Table 2). Interrelationships among the predicted and actual values are described as the following Eq. (2):

**Table 2:** The level of factors, their codes, and actual values involved in central composite design

Code	Independent variables	Low (-1)	Central (0)	High (+ 1)
**A**	pH	6.0	7.0	8.0
**B**	Yeast extract (g L^− 1^)	3.86	7.73	11.25
**C**	NaCl (g L^− 1^)	125	200	275
**D**	Inoculum volume	1	5.5	10
**E**	Incubation time (days)	3	6	9

2 $${X_i} = {X_i} - {X_{cp}}/\Delta {X_i}i = 1,2,3,{\text{ }}......k$$

where X_i_ is the dimensionless value of the independent variables, x_i_ is the real value of the independent variables, x_cp._ is the real value of the independent variables at the central point, and Δx_i_ is the step change in the real value of variable i, representing a variation of a unit for the dimensionless value of variable i. carotenoid production was used as a response. The interaction of the independent variables, their relationships, and the responses was calculated using the second-order polynomial model expressed in the following quadratic Eq. (3):

3 $$Y = {\beta _0} + \sum\limits_{i = 1}^k {{\beta _i}{X_i} + \sum {\sum\limits_{i < j = 1}^k {{\beta _{ii}}{X_i}{X_j} + \sum\limits_{i = 1}^k {{\beta _{ij}}X_i^2} } } }$$

Where Y is the predicted response, β_0_ is the intercept term, β_i_ is a linear coefficient, β_ii_ is a quadratic coefficient, β_ij_ is the interactive coefficient, and X_i_ and X_j_ represent the independent variables in the form of predicted values. Three-dimensional (3D) response surface graphs were plotted by varying the concentrations of the two factors while keeping the concentrations of the other factors at zero. The optimization of carotenoid production was further elucidated by validating the responses obtained under optimized medium conditions. The best medium constitution obtained using RSM was named the ATCC 1176R medium.

### Batch fermentation

To validate the optimized fermentation medium for the large-scale production of carotenoid, the cultivation strain MBLA0099 was cultured in a 7.0-L jar fermenter (KoBioTech, Incheon, South Korea) with a 4.2 L working volume. Fermentation was performed using the ATCC 1176R medium, after sterilization in situ. After sterilization, the seed culture was aseptically transferred into a sterile medium and the fermenter was maintained at 37 °C. The effects of aeration and agitation speed were investigated under various combinations of conditions, including agitation speeds of 200, 500, and 800 rpm. The aeration was flushed continuously using ComVac Oil-less Piston air compressor & vacuum pump HJS 245P (Hanjin Air Tech Co. Lid, Ilsan, South Korea) through a sparger placed at the bottom of the fermenter, and the air was filtered using 0.2 μm polytetrafluoroethylene (PTFE) filter (Sartorius, AG, Germany). The aeration rates were set at 0.5 or 1.0 vvm (volume of air/volume of medium/min). The dissolved oxygen (DO) content of the fermentation medium was assessed using a DO probe and standard two-point calibration using 5% (w/v) sodium sulfite (Samchun, Pyeongtaek, South Korea). Samples from the cell-free broth were withdrawn and changes in sucrose concentration were estimated using a sucrose assay kit (Abcam, Cambridge, UK). Sampling from the culture broth was performed every 12 h, for 3 d, and every 24 h thereafter, for 5 d.

### Antioxidant property of BR extract

#### In vitro antioxidant assay

2,2’-Azinobis-(3-ethylbenzothiazoline-6-sulfonic acid) (ABTS) is typically used with hydrogen peroxide (H_2_O_2_) as the substrate for peroxidase activity [19]. To create the radical cation ABTS*^+^, 2.45 mM potassium persulfate (Duksan, Seoul, South Korea) was mixed in a 1:1 (v/v) ratio with 7 mM ABTS (Sigma-Aldrich, St. Louis, MO, USA) in a 20 mM sodium acetate buffer solution for 14–16 h, in the dark. Ethanol [70% (v/v)] was used to dilute the solution until it had a moderate absorbance of 0.7 ± 0.02 at the wavelength of 734 nm. Following that, 180 µL of the ABTS*^+^ solution + 20 µL of BR extract was reacted for 7 min at room temperature, in the dark. The antioxidant activity of the samples was estimated in terms of the decrease in absorbance at the wavelength of 734 nm, which was expressed as the percentage inhibition of ABTS*^+^ oxidation. The antioxidant activity of the BR extract was compared with that of other antioxidants and commercial C_40_ carotenoids such as 3,5-di-tert-butyl-4-hydroxytoluene [BHT (Kanto, Tokyo, Japan]], ascorbic acid (Sigma-Aldrich, St. Louis, MO, USA), lycopene (Sigma-Aldrich, St. Louis, MO, USA), β-carotene (Sigma-Aldrich, St. Louis, MO, USA), and astaxanthin (Sigma-Aldrich, St. Louis, MO, USA). The antioxidant activity was finally expressed as Trolox (Sigma-Aldrich, St. Louis, MO, USA) equivalent antioxidant capacity (TEAC) and concentration of carotenoids expressed as µg/mL (IC_50_) [20–22].

The ferric-reducing ability of plasma (FRAP) assay is an antioxidant assay based on the formation of the O-phenanthroline-Fe^2+^ complex and its disruption in the presence of chelating agents [23]. A freshly made solution of ferric 2,4,6-tripyridyl-s-triazine (TPTZ-Fe^+ 3^), made by combining 10 mM TPTZ (Sigma-Aldrich, St. Louis, MO, USA), 20 mM FeCl_3_·6H_2_O (Junsei Tokyo, Japan), and 0.3 M acetate buffer, pH 3.6, in a ratio of 1:1:10 (v/v/v), was prepared by dissolving a sample of BR extract in 100% methanol. The assay was carried out for 30 min at room temperature, in complete darkness, after combining 1 mL of FRAP reagent with 100 µL of BRextract. The absorbance at the wavelength of 593 nm was measured to determine its antioxidant activity, as compared to that of other antioxidants, as described above [23, 24]. Data processing was based on standard curves obtained using FeSO_4_·7H_2_O solution. The antioxidant activity of the BR extract was expressed as TEAC.

The Fenton reaction in the plasmid DNA assay involves the formation of hydroxyl radicals from H_2_O_2_ in the presence of ferrous ions. Electrophoretic mobility shifts when the plasmid DNA is damaged by oxidative stress. The supercoiled plasmid DNA then relaxes and takes a linear shape [25]. Fenton reagent was prepared using 10 µL 5% H_2_O_2_ (v/v), 10 µL 100 µM FeCl_2_ (Sigma-Aldrich, St. Louis, MO, USA), and 5 µL 100 mM phosphate buffer (pH 7·4). The assay involved taking 15 µL of pUC19 plasmid DNA 500 µg µL^-1^ (New England Biolabs, Ipswich, MA, USA) and mixing 25 µL of Fenton reagent with it. The reaction mixtures were incubated in the presence or absence of different concentrations (0·25, 0·5, and 1.0 µM) of BR extract dissolved in dimethyl sulfoxide (DMSO) (Sigma-Aldrich, St. Louis, MO, USA). Distilled water was added instead of the H_2_O_2_ solution, while DMSO was added instead of the carotenoid extract in the negative control and blank assays, respectively. Oxidized plasmid DNA was converted to a linear form, and its electrophoretic mobility was compared with that of intact non-oxidized supercoiled plasmid DNA. To confirm the protective properties of the BR extract, other carotenoids (lycopene, β-carotene, and astaxanthin) were tested simultaneously. The assay was incubated at 37 °C for 7 min and stopped by adding 5× loading buffer for agarose gel (Genesta, Stockholm, Sweden). Electrophoresis of the intact damaged plasmid DNA was performed at room temperature on 1.0% (w/v) agarose gels containing Neogreen for 30 min, at 100 mV. Antioxidant capacity was calculated by reducing the degree of DNA relaxation during electroporation. Gel imaging was performed using a ChemiDoc™ MP imager (Bio-Rad, Hercules, CA, USA), while the densitometry of the bands was estimated using ImageJ software (National Institutes of Health, Bethesda, MD, USA).

#### Cellular antioxidant assay

The cytotoxicity and cellular antioxidant activity of the BR extract were evaluated in Caco-2 cells. The cells were obtained from the Korean Cell Line Bank in Seoul, Republic of Korea, and cultured in a growth medium consisting of Dulbecco’s Modified Eagle’s Medium [89% (v/v)], penicillin–streptomycin solution [1% (v/v)], and fetal bovine serum [20% (v/v)]. Caco-2 cells were seeded at a density of 1.5 × 10^4^ cells per well in 96-well plates and incubated at 37 °C, in an atmosphere with 5% CO_2_ and 95% humidity, for 24 h. After incubation, the culture medium was replaced with 200 µL of fresh growth medium containing BR extract solution (0, 3.7, 7.4, 18.5, 37.0, and 74.0 µg mL^− 1^) prepared in DMSO. The final DMSO concentration in the growth medium was 0.1%. After 24 h of treatment with the BR extract, cytotoxicity by Water-Soluble Tetrazolium 1 (WST-1) and LIVE/DEAD™ staining) and Cellular Antioxidant Activity (CAA) assays were conducted. For the WST-1 assay, EZ-Cytox reagent was prepared by mixing it with growth medium at a 1:10 (v/v) ratio, added to each well, and incubated at 37 °C for 3 h. The optical density of the samples was measured at the wavelength of 440 nm using a Varioskan™ LUX multimode microplate reader (Thermo Fisher Scientific Inc., Waltham, MA, USA). In addition, LIVE/DEAD™ staining solution (2 µM of calcein-AM and 4 µM of ethidium homodimer-1) was added to each well and incubated at 37 °C, for 30 min. Stained cells were observed by means of fluorescence microscopy. CAA was estimated using 2’,7’-dichlorofluorescein diacetate (DCF-DA) staining, where 25 µM of DCF-DA solution was applied to BR extract-treated Caco-2 cells and incubated for 45 min. Subsequently, the cells were washed with phosphate-buffered saline (PBS) and exposed to 0.3 mM of tert-butyl hydroperoxide (TBHP) for 1 h. Expression of the fluorescent product (DCF) was measured using microplate spectrophotometry (Ex/Em = 495/535 nm) and fluorescence microscopy. CAA was calculated using the following formula (4):

4 $$CAA{\text{ }}value{\text{ }}(\% ) = [1 - ({A_{treatment}} - {A_{NC}})/({A_{PC}} - {A_{NC}})] \times 100$$

Where A_treatment_ represents cells treated with DCFH-DA and PBS with TBHP and BR extract samples, A_NC_ represents cells treated with DCFH-DA and PBS without TBHP (as a negative control), and A_PC_ represents cells treated with DCFH-DA and PBS with TBHP (as a positive control). The procedures followed the standard protocols described by Chen et al. [26] and Wolfe and Liu [27].

#### In vivo antioxidant assay

The in vivo antioxidant activity of BR extract was assessed in *Caenorhabditis elegans* [28]. Cultivation and synchronization of the worms were performed as described by Rathor et al. [29]. The BR extract was dissolved in DMSO and added to the nematode growth medium (NGM). To compare the antioxidant effects of other commercial C_40_ carotenoids, lycopene, β-carotene, and astaxanthin were added under the same conditions. To measure *C. elegans* survival rates after exposure to oxidative stress, nematode eggs were synchronized and hatched on NGM agar plates with *Escherichia coli* OP50-1, in the presence or absence of each carotenoid (3 µM). DMSO-treated medium without carotenoids was used as the negative control. After 5 d of growth at 20 °C, the worms were transferred to fresh NGM plates containing H_2_O_2_ (3 mM) and left for 5 h. The nematodes were then washed, following which their viability was measured. The worms were considered dead when they no longer responded to prodding or moved.

### BR extraction and quantification

The culture broth was centrifuged at 4 °C and 12,000 × *g* for 5 min to harvest wet cell pellets and treated with acetone/methanol (7:3, v/v) solvent. Following that, the pellets were incubated at 37 °C and 180 rpm for 2–3 h, in the dark, to completely decolorate of the pellets. The mixture of pellets and organic solvent were then centrifuged at 4 °C and 12,000 × *g* for 15 min, to obtain the organic-solvent extracts. The extracts were sufficiently evaporated at 50 °C using smart evaporator C1 (BioChromato, San Diego, CA, USA) and redissolved in 100% methanol to obtain methanolic carotenoid extracts. The methanolic carotenoid extracts were subsequently filtered through a 0.2-µm pore size cellulose acetate membrane (GVS Korea Ltd., Namyangju, South Korea) to subduct salts and other residues. The filtered carotenoid extract was stored at -20 °C, in the dark. In the methanolic extracts, the total carotenoid concentration in the crude carotenoid extract was calculated using Eq. (5):

5 $$Carotenoid{\text{ }}concentration(\mu g/mL) = ({A_{494}} \times {10^6})/(\varepsilon _{1cm}^{1\% } \times {10^2})$$

Absorbance values at the wavelength of 494 nm were measured using a Shimadzu spectrophotometer UV-1200 (Shimadzu, Kyoto, Japan) and used to compute the carotenoid concentration using the extinction coefficient (ε) for BR extract in methanol (2,660 g %^–1^ cm^–1^) [21, 30].

### Statistical analysis

All results are presented as mean ± standard deviation (*n* = 3). Data are presented as the average of three independent experiments. Analysis of variance with Tukey’s *post-hoc* test was performed using Prism 5 software (GraphPad). The results are represented as mean with standard deviation. Statistical significance has been indicated as *p < 0.05, **p < 0.01, and ***p < 0.001. NS indicates that the experimental groups did not differ significantly.

## Results and discussion

### Microbial growth and carotenoid production

Cell growth and total carotenoid production by the strain MBLA0099 in basal ATCC 1176 medium are shown in supplementary material. carotenoid production increased rapidly and peaked after 6 d of cultivation. In the basal ATCC1176 media, there was no significant difference in BR production from the 6–10 d. Thus, subsequent experiments on carotenoid productivity were conducted for 6 d. The maximum carotenoid production of strain MBLA0099 under the initial conditions was 0.496 mg L^–1^.

### Medium optimization using the conventional method

**Fig. 1:**
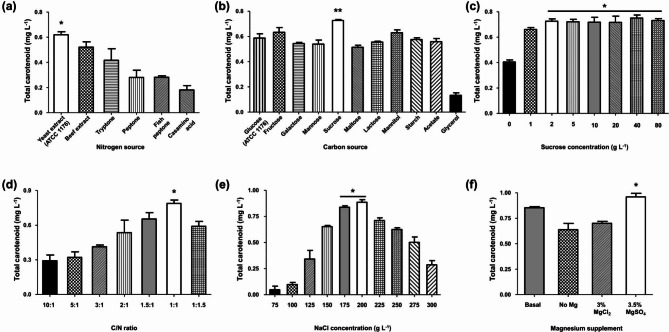
Optimization of total carotenoid production by one factor at a time approach. Effect of **(a)** nitrogen sources **(b)** carbon sources **(c)** sucrose concentration **(d)** C/N ratio **(e)** NaCl concentration **(e)** magnesium supplementation, on total carotenoid production under shaken flask cultivation at 37 °C, 200 rpm for 6 d. Asterisk symbols (* and **) indicate significant differences between others (*p < 0.05 and **p < 0.01, respectively)

The OFAT optimization approach was successfully employed to analyze the impact of several parameters, including carbon and nitrogen sources, C/N ratio, NaCl concentration, and magnesium supplementation, on carotenoid production in strain MBLA0099. Replacing 5 g L^–1^ yeast extract (normally included in basal ATCC 1176 media) with other nitrogen sources resulted in significantly decreased production levels of total carotenoid (Fig. 1a), while replacement with inorganic sources did not induce cell growth (data not shown). The nutritional demands of most haloarchaea are known to include amino acids [31, 32]. Therefore, it was predicted that complex nitrogen sources were preferred for cell growth and secondary metabolite production by the strain MBLA0099, similar to that observed in case of other haloarchaea.

The highest production level of total carotenoid was 1.33-fold (0.658 mg L^–1^) when 1 g L^–1^ glucose was replaced with 1 g L^–1^ sucrose (Fig. 1b). The cell growth of strain MBLA0099 increased by 1.4-fold when glucose was replaced with sucrose (3.32–4.73 g L^–1^ dry cell weight). Strain MBLA0099 contains various genes involved in sucrose uptake and degradation pathways, such as sucrose uptake & hydrolysis, semiphosphorylative Entner-Doudoroff (spED) pathway, modified Embden-Meyerhof-Parnas (EMP) pathway, common spED/EMP shunt, and gluconeogenesis (see supplementary material) [33–35]. Therefore, the accumulation of carotenoid was associated with changes in the suitable carbon source, as an effect of increased cell growth.

The efficiency of total carotenoid production was > 0.717 mg L^–1^ when the initial sucrose concentration was > 2 g L^–1^ (Fig. 1c). The production of carotenoid was not statistically significant in the range of 2–80 g L^–1^ sucrose concentration. The addition of 2 g L^–1^ sucrose resulted in approximately 10% higher carotenoid production (0.725 mg L^–1^) than that observed in the culture with 1 g L^–1^ sucrose (Fig. 1c). An increase in sucrose concentration not only stimulates cell growth but also induces carotenogenesis. This is attributed to the fact that the carbon released during sucrose metabolism serves as the primary component in the molecular structure of carotenoids [36]. However, excess sucrose concentration above a certain level did not require the initial growth phase for high carotenoid production by strain MBLA0099.

With respect to the different C/N ratios, a pattern was identified: when the level of the initial yeast extract was increased from 10:1 (0.77 g L^–1^ of yeast extract) to 1:1 (7.73 g L^–1^ of yeast extract), the total carotenoid production by strain MBLA0099 increased from 0.289 to 0.816 mg L^–1^ (Fig. 1d). However, an initial C/N ratio of 1:1.5 (11.6 g L^–1^ of yeast extract) indicated that high-level yeast extract may be associated with a negative effect on carotenoid production. In particular, the addition of excess yeast extract at a C/N ratio of more than 1:2 to the culture medium failed to induce growth and was detrimental to strain MBLA0099 (data not shown). This result showed that a C/N ratio of 1:1 could be used to carry out mass production of carotenoid and the moderate C/N ratio increased nearly 1.12 times more than untuned (Fig. 1c). A previous study on the effect of the C/N ratio on the growth and pigmentation levels of haloarchaea suggested that BR production by *Haloferax mediterranei* showed the best results with a low C/N ratio (0·5% glucose and 100 mM nitrate, nearly a 1.4:1 of C/N ratio) [37]. The optimal C/N ratio for carotenoid production can vary among different species of haloarchaea, and even within a particular species, it can depend on specific environmental conditions and growth phases. Therefore, understanding and optimizing the C/N ratios involved in growth should be explored to maximize carotenoid production in haloarchaea.

Considering that approximately 15% NaCl (w/v) was added to the basal ATCC 1176 medium, supplying an additional 57.5% NaCl was advantageous for total carotenoid production by strain MBLA0099 (Fig. 1e). The production of carotenoid was more favorable in a medium containing between 20% and 22·5% NaCl than at any of the other concentrations. Therefore, a 20% NaCl concentration was tentatively selected by considering carotenoid production (0.880 mg L^–1^) (Fig. 1e). Growth and carotenoid production decreased at NaCl concentrations < 12.5%, because low NaCl concentrations resulted in cell lysis [38]. NaCl concentrations > 25% caused a decrease in the DO of the broth medium and decreased cell growth, indicating a negative effect on carotenoid production by haloarchaea.

Haloarchaea generally require Mg ions for growth and cell division [39, 40]. High carotenoid production by strain MBLA0099 was observed in the absence of magnesium chloride. However, in the presence of magnesium sulfate, the production of carotenoid increased (Fig. 1f). A significantly high production level of BR (0.959 mg L^–1^) was achieved using a modified medium containing only magnesium sulfate as the magnesium supplement. Upon increasing the concentration of magnesium sulfate in the culture medium, the proportion of carotenoid to the total carotenoids produced by archaea increases, up to a certain level of magnesium concentration [41]. However, carotenoid production was similar even when up to 12% magnesium sulfate was added (data not shown). In haloarchaea, carboxyl groups derived from glycoproteins, featuring high contents of negatively charged acidic amino acids such as aspartate and glutamate, along with sulfate groups, bind to sodium ions abundant in the environment. Upon addition of magnesium sulfate, this binding mechanism can help stabilize cells and influence carotenoid production as cells grow [40]. The addition of magnesium chloride as a magnesium supplement to the medium appeared to have effects that were not significantly different from those observed in the absence of magnesium (Fig. 1f).

### Optimization of total carotenoid production using PBD

The central point of the PBD was established based on the OFAT results. PBD analysis was used to identify the factors that significantly affected total carotenoid production by strain MBLA0099. The PBD matrix and results are listed in Table 3. The effects of the variables on the significant responses according to the *t*-test are shown in supplementary materials. The regression analysis and analysis of variance (ANOVA) for the PBD are shown in Table 4. The coefficient of determination (R^2^) and adjusted R^2^ demonstrated that 99.96% and 99.87% of the variability in the responses was explained by the model (> 90% is usually desired). The ANOVA results revealed that all the variables in this model had a low *p*-value of < 0.039 (Table 2). In particular, pH, yeast extract, NaCl, inoculum volume, and incubation time had a more significant impact (*p* < 0.001) on carotenoid production than the other factors (Table 4 and see supplementary material). Regression analysis resulted in the following first-order polynomial model (6):

**Table 3:** Plackett-burman design matrix of independent variables and their corresponding predicted and actual values of total carotenoid production under shaken flask cultivation at 37 °C, 200 rpm. The coded factors were X_1_ = sucrose, X_2_ = yeast extract, X_3_ = MgSO_4_·7H_2_O, X_4_ = pH, X_5_ = incubation time, X_6_ = inoculum volume, X_7_ = KCl, X_8_ = CaCl_2_·6H_2_O, and X_9_ = NaCl

Run	Coded factor												Total carotenoid (mg L^− 1^)		
	X_A_	X_B_	X_C_	X_D_	X_E_	X_F_	X_G_	X_H_	X_I_			Predicted		Actual	
**1**	+ 1	-1	+ 1	-1	-1	-1	+ 1	+ 1	+ 1			0.139		0.147	
**2**	+ 1	+ 1	-1	+ 1	-1	-1	-1	+ 1	+ 1			0.357		0.350	
**3**	-1	+ 1	+ 1	-1	+ 1	-1	-1	-1	+ 1			0.023		0.023	
**4**	+ 1	-1	+ 1	+ 1	-1	+ 1	-1	-1	-1			0.620		0.620	
**5**	+ 1	+ 1	-1	+ 1	+ 1	-1	+ 1	-1	-1			0.233		0.237	
**6**	+ 1	+ 1	+ 1	-1	+ 1	+ 1	-1	+ 1	-1			0.086		0.086	
**7**	-1	+ 1	+ 1	+ 1	-1	+ 1	+ 1	-1	+ 1			0.504		0.504	
**8**	-1	-1	+ 1	+ 1	+ 1	-1	+ 1	+ 1	-1			0.571		0.564	
**9**	-1	-1	-1	+ 1	+ 1	+ 1	-1	+ 1	+ 1			0.951		0.955	
**10**	+ 1	-1	-1	-1	+ 1	+ 1	+ 1	-1	+ 1			0.395		0.387	
**11**	-1	+ 1	-1	-1	-1	+ 1	+ 1	+ 1	-1			0.045		0.045	
**12**	-1	-1	-1	-1	-1	-1	-1	-1	-1			0.102		0.102	
**13**	0	0	0	0	0	0	0	0	0			0.921		0.955	
**14**	0	0	0	0	0	0	0	0	0			0.921		0.936	
**15**	0	0	0	0	0	0	0	0	0			0.921		0.929	

**Table 4:** Regression analysis and analysis of variance (ANOVA) for the experimental results of the Plackett-Burman design first-order model response total carotenoid production. The coded factors were X_1_ = sucrose, X_2_ = yeast extract, X_3_ = MgSO_4_·7H_2_O, X_4_ = pH, X_5_ = incubation time, X_6_ = inoculum volume, X_7_ = KCl, X_8_ = CaCl_2_·6H_2_O, and X_9_ = NaCl. DF means degree of freedom

Factor	df	Standard error	Coefficient estimation	Sum of squares	Mean of squares	F value	*p*-value
**Model**	**10**		**0.33500**	**1.77045**	**0.177045**	**1169.90**	**0**
X_1_ (Sucrose)	1	0.0123018	-0.03050	0.01116	0.011163	73.76	0.001
X_2_ (Yeast extract)	1		-0.12750	0.19508	0.195075	1289.04	0***
X_3_ (MgSO_4_·7H_2_O)	1		-0.01100	0.00145	0.001452	9.59	0.039
X_4_ (pH)	1		0.20333	0.49613	0.496133	3278.41	0***
X_5_ (Incubation time)	1		0.04033	0.01952	0.019521	129.00	0***
X_6_ (Inoculum volume)	1		0.09783	0.11486	0.114856	758.96	0***
X_7_ (KCl)	1		-0.02100	0.00529	0.005292	34.97	0.005
X_8_ (CaCl_2_·6H_2_O)	1		0.02283	0.00626	0.006256	41.34	0.004
X_9_ (NaCl)	1		0.05933	0.04225	0.042245	279.15	0***

6 $$\displaylines{Carotenoid{\text{ }}production{\text{ }}\left( {mg{\text{ }}{L^{ - 1}}} \right) = {\text{ }}0.33500{\text{ }} - {\text{ }}0.03050{X_1} - \cr 0.12750{X_2} - 0.01100{X_3} + 0.20333{X_4} + 0.04033{X_5} + \cr 0.09783{X_6} - 0.02100{X_7} + 0.02283{X_8} + 0.05933{X_9} \cr}$$

Where X_1_ = sucrose, X_2_ = yeast extract, X_3_ = MgSO_4_·7H_2_O, X_4_ = pH, X_5_ = incubation time, X_6_ = inoculum volume, X_7_ = KCl, X_8_ = CaCl_2_·6H_2_O, and X_9_ = NaCl are variables in this study. The coefficients of the selected factors that had a significant effect on the carotenoid yield indicated that all factors had critical effects on the overall production. Therefore, the most crucial variables (*p* < 0.001) with positive effects were further investigated using the CCD experiment, to determine the optimal range of factors and their interactive effects. Accordingly, the most crucial variables (pH, yeast extract, NaCl, incubation time, and inoculum volume) were further investigated using CCD, based on the results of the PBD analysis.

### Optimization of total carotenoid production using CCD

Based on the PBD results, RSM was performed to optimize the medium composition and physical factors to enhance carotenoid production using CCD. The experimental design runs for the respective and actual yields of each response are listed in Table 5. The results of this design were similar to the values predicted for carotenoid production. The adequacy and fitness of carotenoid production was determined using ANOVA, and the regression coefficients and interactive effects of all factors are shown in Table 6, with confidence intervals (*p* < 0.05). The *p*-values were used to identify the significance of each coefficient and pattern of interactive effects between variables. The estimated coefficient and *p*-values confirmed the terms of significant variables and interactions between variables. The effectiveness was proportional to the value of a single factor. The quadratic term is proportional to the square of the value of the factor and indicates its curvature. The interactive term is proportional to the product of the values of one factor and another. A (pH), D (inoculum volume), and E (cultivation time) were highly effective in this design. A quadratic interaction of variables was determined, as B × B (yeast extract × yeast extract) and C × C (NaCl × NaCl) were significant with *p*-values < 95% (*p* < 0.05). The *p*-values demonstrated that among the tested interactive variables, A × B (pH × yeast extract), A × D (pH × inoculum volume), A × E (pH × incubation time), B × C (yeast extract × NaCl), B × D (yeast extract × inoculum volume), and B × E (yeast extract × incubation time) were significant model terms for carotenoid production. Regression analysis results in the following second-order response model (7):

**Table 5:** Central composite design matrix of independent variables and their corresponding predicted and actual values of total carotenoid production under shaken flask cultivation at 37 °C, 200 rpm. A: pH; B: yeast extract; C: NaCl; D: inoculum volume; E: incubation time

Run	Independent variables						Total carotenoid (mg L^− 1^)			Run		Independent variables						Total carotenoid (mg L^− 1^)		
	A	B	C	D	E	Predicted		Actual			A		B	C	D	E	Predicted		Actual	
**1**	-1	-1	-1	-1	-1	0.139		0.086	**25**		-1		-1	+ 1	-1	+ 1	0.094		0.034	
**2**	+ 1	-1	-1	-1	-1	0.410		0.406	**26**		+ 1		-1	+ 1	-1	+ 1	0.711		0.801	
**3**	-1	+ 1	-1	-1	-1	0		0	**27**		-1		+ 1	+ 1	-1	+ 1	0.083		0	
**4**	+ 1	+ 1	-1	-1	-1	0.308		0.301	**28**		+ 1		+ 1	+ 1	-1	+ 1	0.808		0.872	
**5**	-1	-1	-1	+ 1	-1	0.139		0.071	**29**		-1		-1	+ 1	+ 1	+ 1	0.038		0.064	
**6**	+ 1	-1	-1	+ 1	-1	0.511		0.485	**30**		+ 1		-1	+ 1	+ 1	+ 1	0.752		0.643	
**7**	-1	+ 1	-1	+ 1	-1	0.026		0.004	**31**		-1		+ 1	+ 1	+ 1	+ 1	0.120		0.135	
**8**	+ 1	+ 1	-1	+ 1	-1	0.508		0.410	**32**		+ 1		+ 1	+ 1	+ 1	+ 1	0.947		1.011	
**9**	-1	-1	+ 1	-1	-1	0.117		0.184	**33**		-1		0	0	0	0	0.586		0.462	
**10**	+ 1	-1	+ 1	-1	-1	0.421		0.474	**34**		+ 1		0	0	0	0	1.135		1.244	
**11**	-1	+ 1	+ 1	-1	-1	0		0.045	**35**		0		-1	0	0	0	0.568		0.620	
**12**	+ 1	+ 1	+ 1	-1	-1	0.414		0.477	**36**		0		+ 1	0	0	0	0.560		0.492	
**13**	-1	-1	+ 1	+ 1	-1	0.053		0.071	**37**		0		0	0	-1	0	0.782		0.680	
**14**	+ 1	-1	+ 1	+ 1	-1	0.459		0.447	**38**		0		0	0	+ 1	0	0.853		0.756	
**15**	-1	+ 1	+ 1	+ 1	-1	0.034		0.038	**39**		0		0	-1	0	0	0.692		0.737	
**16**	+ 1	+ 1	+ 1	+ 1	-1	0.560		0.526	**40**		0		0	+ 1	0	0	0.729		0.883	
**17**	-1	-1	-1	-1	+ 1	0.056		0.071	**41**		0		0	0	0	-1	0.733		0.714	
**18**	+ 1	-1	-1	-1	+ 1	0.639		0.624	**42**		0		0	0	0	+ 1	0.891		0.895	
**19**	-1	+ 1	-1	-1	+ 1	0		0	**43**		0		0	0	0	0	0.857		0.865	
**20**	+ 1	+ 1	-1	-1	+ 1	0.643		0.609	**44**		0		0	0	0	0	0.857		0.846	
**21**	-1	-1	-1	+ 1	+ 1	0.064		0.150	**45**		0		0	0	0	0	0.857		0.835	
**22**	+ 1	-1	-1	+ 1	+ 1	0.748		0.688	**46**		0		0	0	0	0	0.857		0.917	
**23**	-1	+ 1	-1	+ 1	+ 1	0.068		0.071	**47**		0		0	0	0	0	0.857		0.891	
**24**	+ 1	+ 1	-1	+ 1	+ 1	0.850		0.793	**48**		0		0	0	0	0	0.857		0.857	

**Table 6:** Analysis of variance (ANOVA) for the experimental results in the central composite design quadratic model and response regarding to total carotenoid production by strain MBLA0099. A: pH; B; yeast extract; C; NaCl; D; inoculum volume; E; incubation time. df means degree of freedom

Factor	DF	Standard error	Coefficient estimation	Sum of squares	F value	*p*-value
**Model**	**20**	**0.00452**	**-0.424**	**0.394366**	**78.4**	**0**
**A**	1	0.0027	-0.0017	0.180964	719.55	0***
**B**	1		+ 0.0669	0.000038	0.15	0.699
**C**	1		+ 0.02525	0.000927	3.69	0.065
**D**	1		+ 0.00053	0.003047	12.12	0.002**
**E**	1		-0.0172	0.015377	61.14	0***
**A** ^**2**^	1	0.0101	+ 0.00025	0.000001	0	0.956
**B** ^**2**^	1		-0.005743	0.01519	60.4	0***
**C** ^**2**^	1		-0.000698	0.003807	15.14	0.001**
**D** ^**2**^	1		-0.000542	0.000298	1.18	0.286
**E** ^**2**^	1		-0.00136	0.000368	1.46	0.237
**AB**	1	0.0028	+ 0.001306	0.001677	6.67	0.016*
**AC**	1		+ 0.000194	0.000153	0.61	0.443
**AD**	1		+ 0.001	0.001459	5.8	0.023*
**AE**	1		+ 0.004601	0.013717	54.54	0***
**BC**	1		+ 0.000225	0.001244	4.95	0.035*
**BD**	1		+ 0.000389	0.001335	5.31	0.029*
**BE**	1		+ 0.000632	0.00157	6.24	0.019*
**CD**	1		-0.000131	0.000621	2.47	0.128
**CE**	1		+ 0.000171	0.000475	1.89	0.181
**DE**	1		+ 0.000034	0.000007	0.03	0.87

7 $$\displaylines{Carotenoid{\text{ }}production{\text{ }}(mg{\text{ }}{L^{ - 1}}){\text{ }} = {\text{ }} - 1.625{\text{ }} + {\text{ }}0.015A{\text{ }} + {\text{ }} \cr 0.2557B{\text{ }} + {\text{ }}0.0871C{\text{ }} + {\text{ }}0.0033D - 0.0595E - 0.0006{A^2} - \cr 0.02185{B^2} - 0.002422{C^2} - 0.00219{D^2} - 0.00553{E^2} + \cr 0.00490AB + 0.000708AC + 0.00378AD + 0.01728AE + \cr 0.000840BC + 0.001464BD + 0.002365BE - \cr 0.000488CD + 0.000646CE + 0.000127DE \cr}$$

**Fig. 2:**
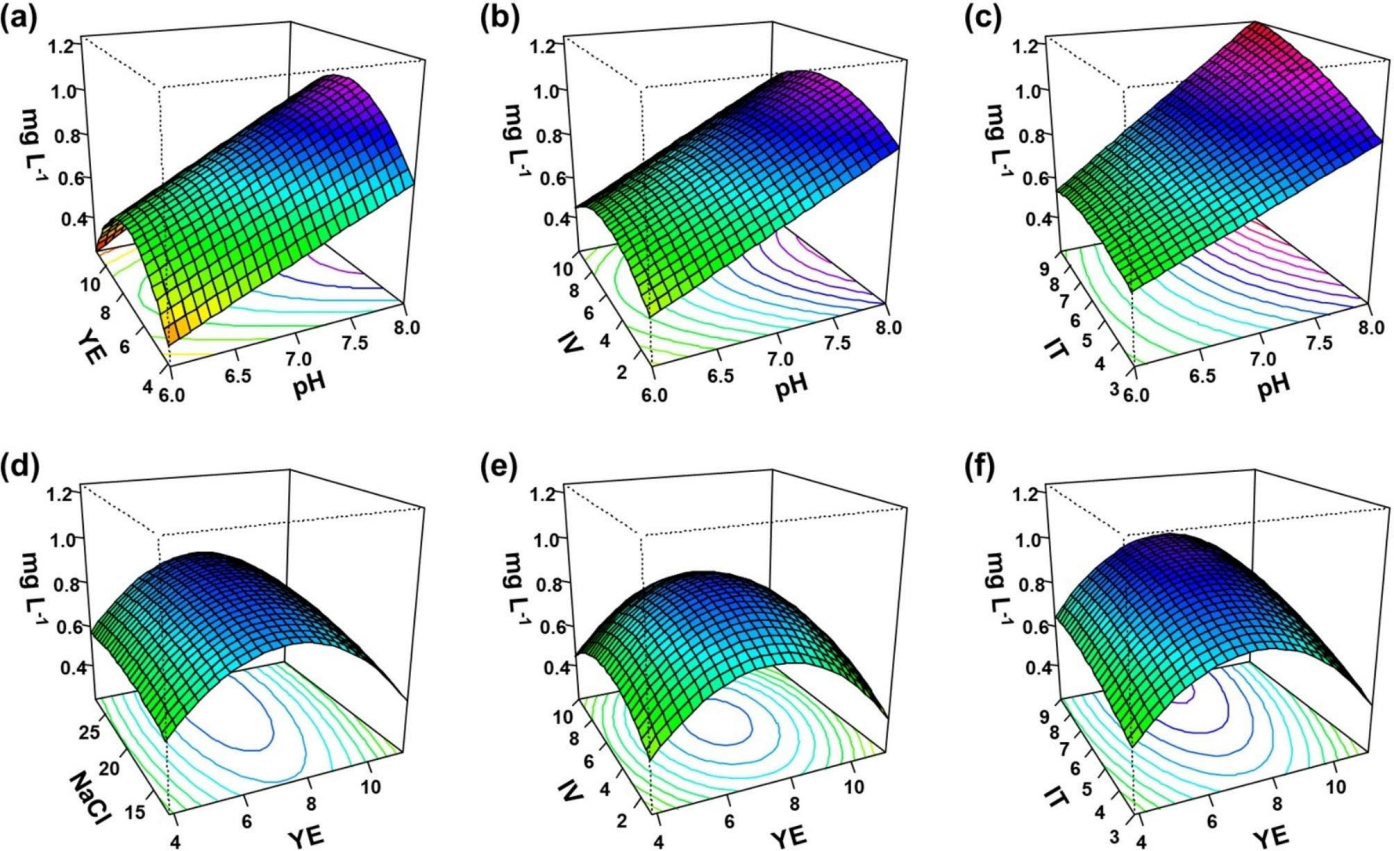
3D response surface plots representing the interactions of various variable pairs on total carotenoid production: **(a)** pH and yeast extract **(b)** pH and inoculum volume **(c)** pH and incubation time **(d)** yeast extract and NaCl **(e)** yeast extract and inoculum volume **(f)** yeast extract and incubation time. YE: yeast extract; IV: inoculum volume; IT: incubation time

The fit of the model was 98.31% and 97.05% variability in the R^2^ and adjusted R^2^ of the response, respectively (> 90% is usually desired). Consequently, the 3D response surface plot showed an interaction between the significant variables (Fig. 2). Carotenoid production improved when the pH and incubation time were maximized, with an increase in the interaction effect of yeast extract concentration, NaCl concentration, and inoculum volume until it approached the center point. Based on these results, an optimized culture medium was composed of 7.96 g L^–1^ yeast extract and 210 g L^–1^ NaCl, pH 8.0, with 8.7% inoculum volume, and cultivation carried out for 9 d. The maximum predicted response for total carotenoid production by MBLA0099 was obtained and confirmed experimentally. The maximum production of carotenoid according to the optimal RSM conditions was 1.233 mg L^–1^, whereas the predicted value was 1.269 mg L^–1^ based on the results of the RSM regression study. This result indicates that CCD is effective in optimizing medium composition and culture conditions where several variables could affect carotenoid production. In total, carotenoid production increased 2.48-fold, as compared to that observed in the basal ATCC1176 medium in the flask culture system.

Upon optimization of carotenoid production by *Hfx. alexandrinus* and *Hfx. mediterranei*, the optimal conditions for production of canthaxanthin by *Hfx. alexandrinus* were found to be 37 °C, pH 7.2, and 250 g L^–1^ NaCl (2.06 mg g^–1^ DCW) based on the OFAT approach, while the maximum carotenoid production of *Hfx. mediterranei* were observed at 36.81 °C, pH 8.96, and 120.3 g L^–1^ of NaCl (3.74 mg L^–1^) using the CCD approach [42, 43]. The optimal conditions for total carotenoid production by *Halorubrum* sp. SH1 using OFAT have been reported as 250 g L^–1^ NaCl, 37 °C, 550 rpm, and a light intensity of 200 µE m^–2^ s^–1^ (25 mg L^–1^), while those for CCD-based total carotenoid production by *Halorubrum* sp. TBZ126 have been reported as 31 and 32 °C, pH 7.51 and 7.94, and 183.3 g L^–1^ and 205.5 g L^–1^ of NaCl (11.71 mg L^–1^) [30, 44]. The variables associated with carotenoid production in haloarchaea differ considerably from those reported in other studies. Thus, these effects should be studied for specific haloarchaea.

### Carotenoid production in a laboratory-scale fermenter using optimal conditions

Fermentations under optimal RSM conditions were carried out in various combinations to confirm the possibility of using an optimized fermentation medium for total carotenoid production at a larger fermenter scale. In the strain MBLA0099, carotenoid production was maximized in the early stationary or middle death phases under all conditions. DO concentrations were maintained at > 60% saturation without any other manipulation and the pH was scored in the range of 7.9–8.5 under all conditions of fermentation.

Carotenoid production was observed to be almost the same and significantly lower at the two aeration levels, with the operating agitation speed maintained at 200 rpm (see supplementary material). Limited agitation resulted in lower cell growth, longer lag phase, and shorter log phase, as compared to those observed at 500 and 800 rpm. Sucrose consumption was nearly 50% at 200 rpm agitation, during an incubation period of 5 d. Lower agitation speeds may have been insufficient to achieve a high amount of carotenoid. Additionally, insufficient sucrose metabolism could be the reason for low carotenoid production. The pH was scored in the range of 7.9–8.1 under 200 rpm conditions of fermentation. The DO values decreased rapidly when the strain entered the exponential phase (36–48 h), but then recovered to a certain level.

Extreme agitation resulted in a shorter lag phase and reached the stationary phase at 60 h; the remaining sucrose concentration also reached 0% at 60 h (see supplementary material). However, higher agitation speeds aggravated the cells, easily damaged them, and caused cell lysis. At 800 rpm, the lysed cells were detected in the culture supernatant withdrawn by means of centrifugation after 2 d of fermentation. These results revealed that excessive agitation speed induces a shearing effect on the cells and may contribute negatively to cell stability and carotenoid production. DO concentrations were maintained at > 40% saturation without any other manipulation. pH was observed to increase to approximately 8.8–8.9 at the end of the fermentation for both 0.5 and 1.0 vvm cultures under 800 rpm condition. Although, it was not fully remarked, the elevation in pH predicted by the release of internal substances resulting from cell lysis.

**Fig. 3:**
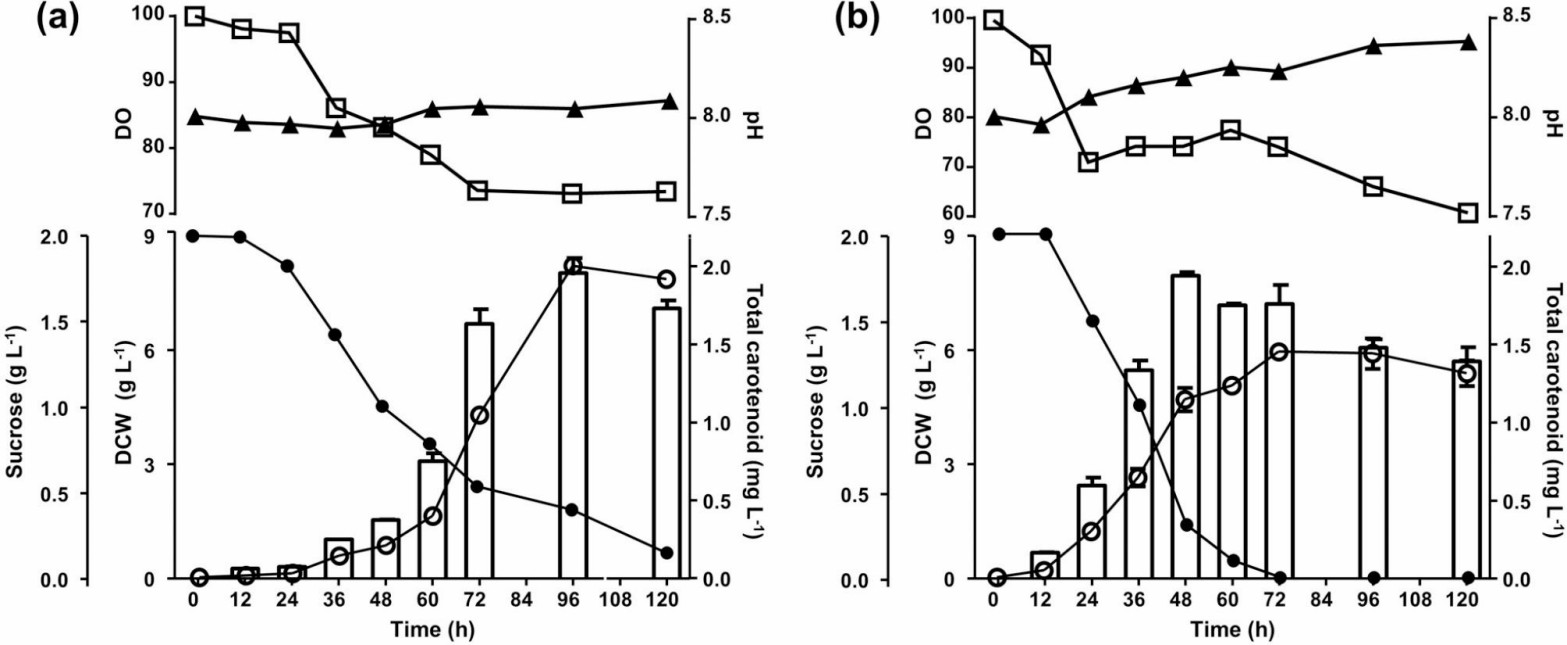
Time-course profiles on lab-scale 7 L fermenter showing 500 rpm agitation with **(a)** 0.5 vvm and **(b)** 1.0 vvm aeration with corresponding cell density (white bar), total carotenoid production (open circle), residual sucrose concentration (closed circle), dissolved oxygen (open square), and pH (closed triangle)

This study indicated that an agitation speed of 500 rpm was optimal for strain MBLA0099 to produce carotenoid (Fig. 3). In the early fermentation stage between 0 and 36 h, cell growth and carotenoid production were slower at 500 rpm and 0.5 vvm than at 800 rpm and 1.0 vvm. After 48 h, strain MBLA0099 showed a longer log phase and a sharp increase in cell growth, with the maximum carotenoid production reaching 1.996 mg L^–1^ at 96 h and then decreasing gradually (Fig. 3a). The DO concentration decreased by 73% at the end of the log phase (96 h) and was consistently maintained thereafter. Sucrose consumption was approximately 80% during the fermentation period of 5 d. Thus, an appropriate agitation speed and aeration rate were the most effective for carotenoid production by strain MBLA0099. DO concentrations were maintained at > 60% saturation without any other manipulation and the pH was scored in the range of 7.9–8.4.

Before 48 h, there was an extreme increase in cell growth after aeration at 1.0 vvm. After 48 h, however, the cell growth was repressed and a significant increase in carotenoid production was observed. Carotenoid production peaked at 72 h (1.444 mg L^–1^) (Fig. 3b). The remaining sucrose concentration reached 0% at 72 h and DO concentration declined by 74.5% at the end of the log phase (48 h). The reason for the DO concentration being lower than that observed at the 0·5 vvm aeration rate could be that the increase in cell growth rate according to the increase in sucrose consumption affected the total DO concentration. This result suggests that a high aeration rate would provide fast cell growth and sucrose consumption, but the mass production of total carotenoid by strain MBLA0099 did not necessarily require fast cell growth and high cell density in the fermentation system. Under fermentation conditions, cell growth kinetics and stoichiometric analysis calculations were presented with respect to variations in agitation and aeration (Table 7). The values were determined based on the optimal time for carotenoid production in each fermentation conditions.

**Table 7:** Effects of agitation speed and aeration rate on the total carotenoid production, cell growth, and specific carbon consumption rate under batch-fermentation by strain MBLA0099

Agitation (rpm)	Aeration (vvm)	Optimal growth time (h)	Total carotenoid production			Cell growth			Specific carbon uptake rate (g g^− 1^ h^− 1^)
			Concentration* (mg L^− 1^)	Yield* (mg g^− 1^)	Productivity* (mg L^− 1^ d^− 1^)	DCW* (g L^− 1^)	Yield* (g g^− 1^)	Specific growth rate (h^− 1^)	
200	0.5	120	0.679	1.108	0.222	3.916	6.389	0.021	0.0023
	1		0.896	1.248	0.250	3.255	4.533	0.021	0.0023
500	0.5	96	1.996	1.252	0.492	8.028	5.036	0.028	0.0042
	1		1.444	0.722	0.359	6.064	3.032	0.030	0.0065
800	0.5	60	1.020	0.510	0.408	4.471	2.236	0.024	0.0099
	1		0.920	0.460	0.368	3.744	1.872	0.020	0.0105

*The values were calculated based on each optimal growth time

Consequently, an optimized fermentation with 500 rpm agitation speed supported the production of carotenoid, and at 0.5 vvm aeration speed, carotenoid production increased by 3.96-fold, as compared to that in the un-optimized flask condition. The RSM-based optimal cultivation time was 9 d, but because of the fermentation growth and level of carotenoid production by strain MBLA0099, cultivation for 96 h was optimal for carotenoid production (Fig. 3a). At 96 h in a 7-L batch fermentation, a DCW (8.03 g L^–1^) and maximal carotenoid production (1.996 mg L^–1^) were attained. Haloarchaea are generally aerobes; therefore, direct oxygen supply, such as aeration and agitation, affects the growth rate of strain MBLA0099. Compared to the cultivation of basal ATCC1766, the total carotenoid productivity increased by 604.5% (from 0.083 to 0.492 mg L^–1^ d^–1^). Therefore, the fermentation system for cultivation of strain MBLA0099 was preferable to the flask culture system for improving carotenoid production from an industrial perspective.

There have been some reports on the optimization of carotenoid production using *Halorubrum* strains. For example, in the flask culture system, Hamidi et al., [44] who approached CCD-based optimization using *Halorubrum* sp. TBZ126, showed 11.71 mg L^–1^ of total carotenoid production for 9 d. Using the OFAT method, De la Vega et al. [30] also found the optimal conditions, including light intensity, for total carotenoid production (25 mg L^–1^) by *Halorubrum* sp. SH1. Although the carotenoid production levels observed in this study are less than those reported in other studies using *Halorubrum* sp., we suggest that the OFAT-based RSM-CCD approach and scale-up fermentation could be applied for industrial production of carotenoids using *Halorubrum* strains, to improve productivity by optimizing the steps. In another study, an open fermentation system with unsterile medium containing *Halorubrum* sp. HRM-150 enhances carotenoid production and is cost-effective because of except sterilization process [45]. Another haloarchaeal strain, *Hfx. mediterranei* ATCC 33,500, exhibited a 92% increase in carotenoid yield in a conceived 2-stage cultivation system [41]. Overall, the desired carotenoid productivity could be achieved by combining various optimization methods using haloarchaea.

### In vitro antioxidant property of BR extract

Three different assays were used to evaluate the antioxidant capacity of the BR extract from MBLA0099. First, the ABTS assay showed that the methanolic BR extract was highly efficient at scavenging free radicals, as compared with the other antioxidants (Table 8). The ABTS assay results, expressed as TEAC, indicated that the antioxidant activity of BR extract was 1.8–8.3-fold times higher than those of β-carotene, astaxanthin, lycopene, ascorbic acid, and BHT. In addition, the IC_50_ of the BR extract was lower than those of BHT, ascorbic acid, and other C_40_ carotenoids. Thus, it can be concluded that the BR extract from strain MBLA0099 showed stronger free radical elimination than the other antioxidants. The IC_50_ values for ABTS radical scavenging ability of carotenoid extracts from Haloarchaea showed different. Within the same genus of *Halorubrum*, *Hrr tebenquichense* strain TeSe-85 and TeSe-86 exhibited values of 7.98 and 4.23 µg mL^–1^, respectively [46]. The extracts from *Hfx. mediterranei*, *Halobacterium salinarum*, *Halococcus morrhuae*, *Haloterrigena* sp. SGH1 showed outstanding scavenging activities (IC_50_ < 0.85 µg mL^–1^) than other haloarchaea [21, 36, 47]. The four *Haloarcula* sp. (HM1, ALT-23, TeSe-41, TeSe-51, and TeSe-89) presented values between 3.89 and 34.72 µg mL^–1^ [36, 48]. While the obtained IC_50_ value (9.8 µg mL^–1^) from this study may not be the highest among previously reported haloarchaeal carotenoid extracts, it has been confirmed to be high in comparison to other haloarchaeal species. The variations observed between assays could be attributed to the composition of each haloarchaeal carotenoid extracts and reacted radical concentrations.

**Table 8:** Antioxidant activity of carotenoid extracted from strain MBLA0099. The antioxidant effects were expressed by TEAC mean ± SD compared to Trolox, ascorbic acid, BHT, and other C_40_ carotenoids

Antioxidant	TEAC (ABTS)		TEAC (FRAP)
	µg/ml	IC_50_	µg/ml
Trolox	1.0	24.5	1.0
BR extract	2.5 ± 0.2	9.8	2.1 ± 0.1
β-carotene	1.2 ± 0.1	18.2	-
Lycopene	1.1 ± 0.2	19.2	-
Astaxanthin	0.3 ± 0.0	81.7	0.2 ± 0.0
Ascorbic acid	1.0 ± 0.0	23.4	0.8 ± 0.1
BHT	0.7 ± 0.1	34.9	0.2 ± 0.0

Second, the TEAC values obtained using the FRAP assay indicated that BR extract from strain MBLA0099 showed considerable antioxidant capacity, nearly 2.1-, 2.7-, 10.5-, and 10.8-fold higher than those of Trolox, ascorbic acid, BHT, and astaxanthin, respectively (Table 8). Since lycopene and β-carotene are relatively hydrophobic, almost no antioxidant effect was detected in the FRAP assay using water-soluble compounds, as compared to that observed in the ABTS assay. This result also demonstrates that BR extract from strain MBLA0099 is an excellent antioxidant that reduces the capacity of iron. *Hrr. tebenquichense* SU10 exhibited a significant antioxidant capacity, measuring 0.31 µM Trolox equivalents/mL of extract, when assessed with a 1000 µM solution of carotenoid extract [49]. Carotenoid extracts from *Halorhabdus utahensis*, depending on the carbon source, demonstrated an average of 1.4 µg mL^–1^ TEAC [50]. The FRAP assay results revealed that carotenoid extracts from *Haloterrigena* sp. SGH1 exhibited an impressive antioxidant capacity, measuring nearly 10.5 µg mL^–1^ TEAC [47]. Similar to the ABTS assay, there were differences in the FRAP activity of carotenoid extracts from various haloarchaea. Notably, the TEAC value of the BR extract from strain MBLA0099 was confirmed to be high at 2.1 µg mL^–1^.

**Fig. 4:**
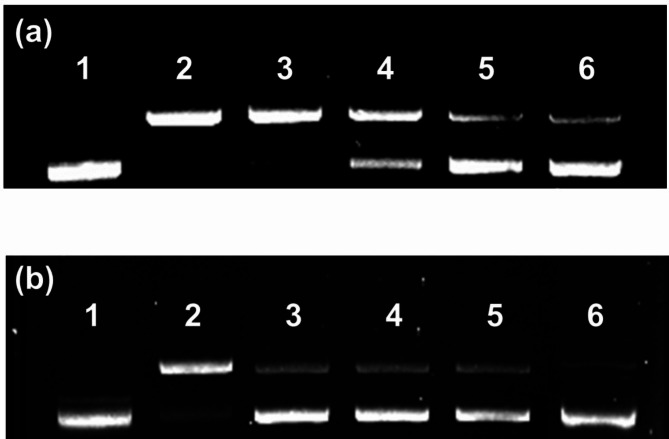
DNA relaxation assay used to evaluate the antioxidant activity of BR extract from strain MBLA0099. **(a)** Dose-dependent antioxidant activity of the bacterioruberin dissolved in DMSO extracted from strain MBLA0099. Lane 1, treated water (as negative control); lane 2, treated Fenton’s reagent (as positive control); lane 3, treated Fenton’s reagent plus DMSO (as blank); lane 4, treated Fenton’s reagent plus 0.25 µM BR extract ; lane 5, treated Fenton’s reagent plus 0.5 µM MBLA0099 extract; lane 6, treated Fenton’s reagent plus 1 µM MBLA0099 extract. **(b)** Antioxidant activity of BR extract from strain MBLA0099 compared to various C_40_ carotenoids. Lane 1, treated water (as negative control); lane 2, treated Fenton’s reagent (as positive control); lane 3, treated Fenton’s reagent plus 3 µM lycopene; lane 4, treated Fenton’s reagent plus 3 µM β-carotene; lane 5, treated Fenton’s reagent plus 3 µM astaxanthin; lane 6, treated Fenton’s reagent plus 0.5 µM BR extract

Third, the protective role of BR extract from strain MBLA0099 against oxidative DNA damage was tested using the plasmid pUC19. Incubation of the supercoiled plasmid DNA with Fenton reagent relaxed the original supercoiled DNA, with shifted electrophoretic mobility (Fig. 4). DNA relaxation was diminished by the addition of BR extract. The protection levels against DNA oxidation were 42%, 64%, and 71% when the concentrations of the BR extract were 0.25, 0.5, and 1 µM, respectively (Fig. 4a). Lycopene, β-carotene, and astaxanthin were found to be insufficient as antioxidants in comparison to BR extract, which displayed a better effect at a 6-fold lower concentration (Fig. 4b). There was a 30%, 32%, 55%, and 78% decrease in DNA relaxation by lycopene, β-carotene, astaxanthin, and the BR extract, respectively, as compared to that in the positive control.

Consequently, BR extract from the strain MBLA0099 exhibited higher antioxidant activity than other commercial C_40_ carotenoids. More number of conjugated double bonds (13) and the presence of four hydroxyl groups may be responsible for these observed outstanding antioxidant properties in vitro [51]. In addition, the increase in antioxidant properties with increasing carotenoid concentrations support the results of the DNA protection test. These results are in agreement with those of previous studies that used haloarchaeal carotenoids. For example, the DPPH radical-scavenging ability of carotenoids extracted from *Haloterrigena thermotolerans* K15 was significantly higher than those of BHT and ascorbic acid [52]. The antioxidant capacity of *Haloterrigena* sp. SGH1 carotenoids is superior to that of BHT and other C_40_ carotenoids (astaxanthin, β-carotene, and lycopene), as observed based on ABTS and FRAP assays, and Fenton reaction on plasmid DNA [47]. The radical scavenging activity of the carotenoid extract from *Haloterrigena turkmenica* was higher than those of BHT, tocopherol, and ascorbic acid [14]. Carotenoid extracts from various haloarchaeal strains, such as *Haloferax* sp., *Halogeometricum* sp., *Haladaptatus* sp., *Haloplanus* sp., and *Halopelagius* sp., are more potent radical scavengers than β-carotene [51]. Haloarchaea-derived carotenoids, especially BR, have better antioxidant activity than conventional antioxidants. Although the antioxidant effects showed slight differences between species, it is expected that natural BR includes geometric isomers, and the ratios of isomer-configured BR are different according to species.

### Cellular antioxidant property of BR extract on caco-2 cells

Chemical antioxidant methods are widely used to screen antioxidant materials. However, these methods have limitations in predicting the antioxidant activity in vivo. One major limitation is that they did not consider cellular uptake, distribution, and metabolism. To address these limitations, cell-based assays have been developed to provide a better biological evaluation of antioxidant activity [26]. The CAA assay measures the ability of a compound to protect cells from oxidative damage. The CAA assay considers the complex processes of cellular uptake, distribution, and metabolism, thus making it a more accurate indicator of the in vivo activity of antioxidants.

**Fig. 5:**
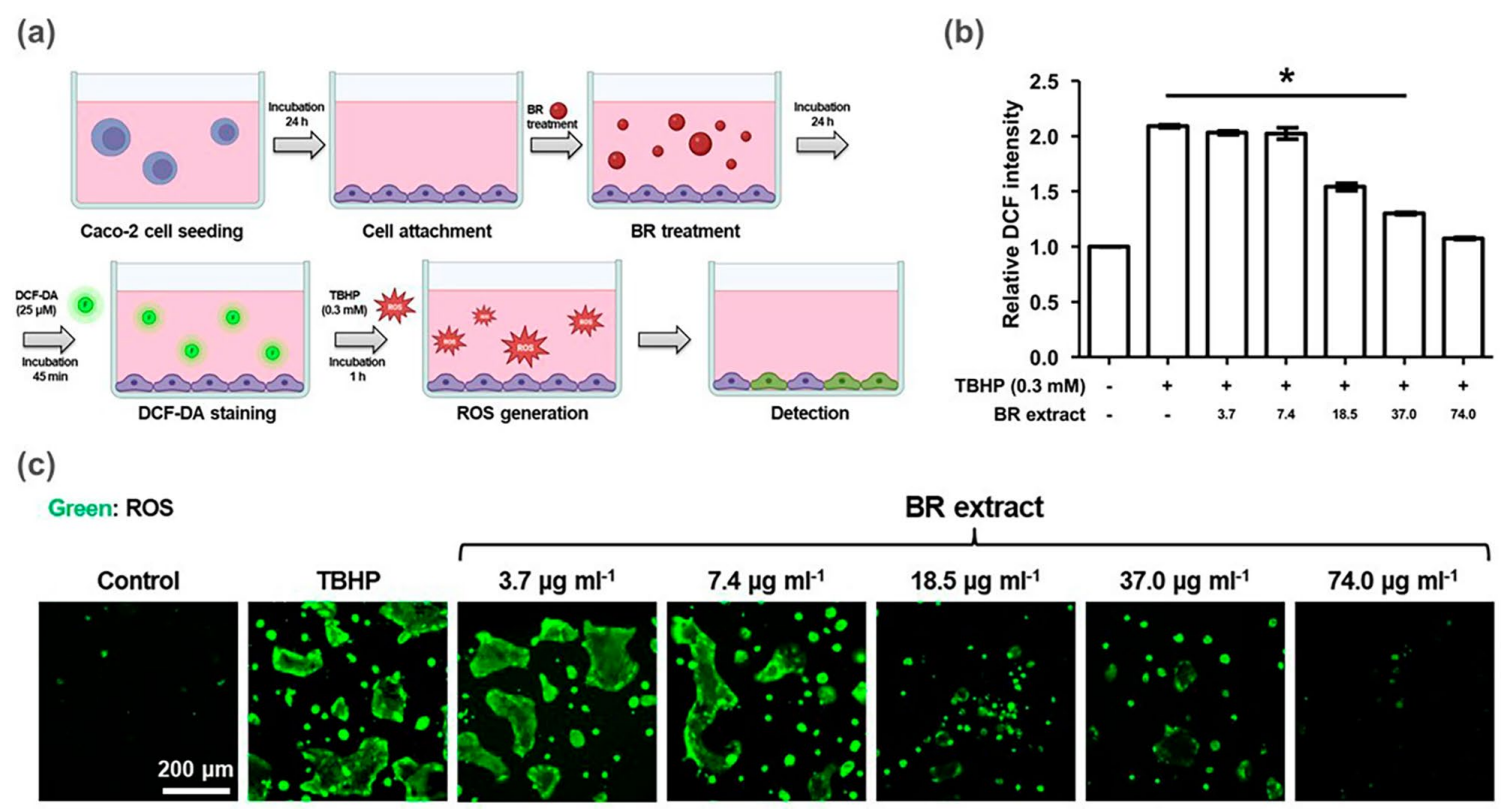
Cellular antioxidant activity of BR extract at different concentrations. **(a)** Scheme of cellular antioxidant activity **(b)** cellular antioxidant activity unit and **(c)** cell morphology. Asterisk symbols (*) indicate significant differences between others (*p < 0.05)

To evaluate the CAA value of a compound accurately, it is important to first investigate its toxicity. Compounds that are toxic to cells may interfere with CAA assays and produce inaccurate results. In this study, when BR extract dissolved in DMSO was tested for its toxicity to cells, it was found that it did not significantly decrease cell viability at concentrations up to 74 µg mL^–1^. The survival rate of the cells was > 98%, indicating that the BR extract was not toxic to the cells (see supplementary material). The results of the CAA assay showed that the cellular antioxidant activity increased with the concentration of the BR extract, with significant differences observed in a dose-dependent manner (*p* < 0.05) (Fig. 5). Compounds that exhibit antioxidant effects in the CAA assay must break peroxyl radical chain reactions at the cell membrane or be taken up by cells to react with intracellular reactive oxygen species (ROS) [27].

In this study, the BR extract demonstrated the greatest protection against peroxyl radicals under physiological conditions in the CAA assay, which agreed with the results of the chemical assays. In summary, the CAA assay provides a better biological evaluation of antioxidant activity, considering cellular uptake, distribution, and metabolism. To ensure accurate results, it is important to investigate the toxicity of compounds before evaluating their CAA values. The results of the CAA assay showed that the BR extract had significant antioxidant activity and was not toxic to the cells, thus highlighting that it is a promising candidate for further evaluation as a potential antioxidant.

### In vivo antioxidant property of BR extract on *C. Elegans*

**Fig. 6:**
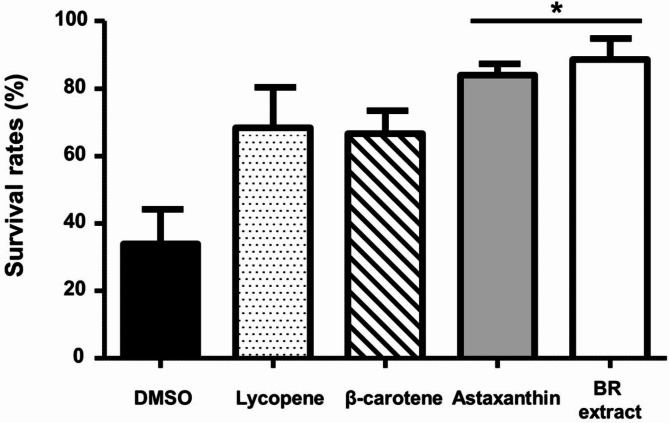
Survival rate of *C. elegans* fed BR extract from strain MBLA0099 compared to other C_40_ carotenoids. More than 100 worms were used in each group. Asterisk symbols (*) indicate significant differences between others (*p < 0.05)

The oxidant effect observed with 2 mM of H_2_O_2_ induced high lethality in the nematodes. Nematodes grown only in DMSO-treated medium without carotenoids showed a survival rate of < 35% upon 5 h of exposure to 2 mM H_2_O_2_. However, treating nematodes with each carotenoid resulted in significant antioxidant effects, which were nearly > 2-fold of those observed in the control group. In particular, the addition of BR extract from strain MBLA0099 to the nematodes resulted in a survival rate of 88.6%, which was ~ 3-fold higher than that observed in nematodes grown on only DMSO-treated medium (Fig. 6). The comparison groups treated with the C_40_ carotenoids lycopene and β-carotene showed survival rates of 68.2% and 66.7%, respectively. Nematodes treated with astaxanthin displayed 84.0% survival, similar to those treated with BR extract from strain MBLA0099 (Fig. 6). Astaxanthin has two hydroxyl and ketone groups in its molecular structure, whereas BR has two hydroxyl groups at each end. Therefore, even though similar to the C_40_ carotenoids, astaxanthin seemed to show effects similar to those of the C_50_ carotenoid BR extract from strain MBLA0099, because of its high values of bio-absorbability, owing to a small hydrophilic property. This result indicates that upon treatment with the same concentration, the BR extract from strain MBLA0099 resulted in the highest survival rate of the nematodes, followed by astaxanthin, lycopene, and β-carotene.

There are reports on the effects of other carotenoids on oxidative stress in *C. elegans*, such as scavenging of free radicals, countering ROS production, expression of antioxidant enzyme genes, modulation of signaling pathways, and reduction of mitochondrial ROS production [53–55]. All these factors affect the lifespan and survival rate of *C. elegans*. Carotenoid extracts from *Haematococcus lacustris* reduce oxidative damage and extend the lifespan extension of *C. elegans* [56]. Mamey (*Pouteria sapota*) and carrot (*Daucus carota*) were found to contain 44.2 µg g^–1^ and 54.7 µg g^–1^ of β-carotene and the carotenoid extracts reduced 20–40% of the oxidative damage in *C. elegans*, respectively [57]. Liu et al. [54] showed that stereoisomeric astaxanthin contributes to the survival rate of worms against oxidative resistance by decreasing ROS levels and upregulating the activity of enzymes related to antioxidative mechanisms. The extract of the red seaweed *Chondrus crispus*, which contains a variety of bioactive components such as lutein and chlorophyll, has been reported to modulate oxidative stress in *C. elegans*, indicating potential stress-alleviating properties [58]. Our study confirmed the antioxidant effect of the C_50_ carotenoid in *C. elegans*, consistent with the findings of previous studies, and is the first study to demonstrate that *C. elegans* fed BR extract from haloarchaea showed enhanced ROS resistance and increased survival rates. Although there is a need for further studies on enzyme activity, gene expression, and RNA-seq analysis of signaling pathways, this finding indicates that the BR extract mainly containing C_50_ carotenoid bacterioruberin from *Hrr. ruber* MBLA0099 may have outstanding antioxidant activity, as compared to other commercial C_40_ carotenoids.

## Conclusions

This study reported enhanced production of the total carotenoid by *Hrr. ruber* MBLA0099 using an OFAT-based RSM-CCD approach and these strategies and fermentation processes achieved increasing productivity. BR showed high antioxidant potential, both in vitro and in vivo and further in vivo experiments are planned to demonstrate the efficacy of BR extract from strain MBLA0099. These results emphasize that carotenoid production by means of microbial biosynthesis is an alternative to conventional chemical synthesis and plant extraction.

### Electronic supplementary material

Below is the link to the electronic supplementary material.

Supplementary Material 1

## Data Availability

All of data in this study are available within the article and its supplementary materials.
