# Supplementary material for: Optimization of bacterioruberin production from Halorubrum ruber and assessment of its antioxidant potential
Source: Microb Cell Fact. 2024 Jan 3;23:2. doi: 10.1186/s12934-023-02274-0 (PMC10762969; doi:10.1186/s12934-023-02274-0)

**Supplementary Table S1**

Gene, location, and function related to sucrose metabolism encoded in genome of strain MBLA0099

| **Pathway** | **Genes** | **Location (bp)** | **Function** |
| --- | --- | --- | --- |
| Sucrose uptake & hydrolysis | Sucrose ABC  transport system | 1,359,754-1,360,758 | Sugar ABC transporter permease |
|  | β-fructofuranosidase (GH32) | 1,364,868-1,367,021 | Sucrose-6-phosphate hydrolase [EC 3.2.1.26] |
|  | Sucrose hydrolase (GH15) | 2,645,171-2,647,297 | Glucan 1,4-α-glucosidase [EC 3.2.1.3] |
| spED pathway | Glucose dehydrogenase | 459,708-460,538 | Glucose 1-dehydrogenase, SDR family oxidoreductase  [EC 1.1.1.100] |
|  | Gluconate dehydratase | 460,579-461,730 | Galactonate dehydratase [EC 4.2.1.6] |
|  | KDG kinase | 458,600-459,556 | Sugar kinase, PfkB domain protein [EC 2.7.1.45] |
|  | KDPG aldolase | 99,160-99,804 | Bifunctional 4-hydroxy-2-oxoglutarate aldolase/  2-dehydro-3-deoxy-phosphogluconate aldolase [EC 4.1.2.14] |
| Modified EMP pathway | FBP aldolase class I | 600,015-600,818 | Fructose-bisphosphate aldolase [EC 4.1.2.13] |
|  | Triose phosphate isomerase | 2,451,047-2,451,691 | Triose-phosphate isomerase [EC 5.3.1.1] |
| Common spED/EMP shunt | GAPDH type I | 644,590-645,642 | Type I glyceraldehyde-3-phosphate dehydrogenase [EC 1.2.1.12] |
|  | Phosphoglycerate kinase | 645,739-646,962 | Phosphoglycerate kinase [EC 2.7.2.3] |
|  | Phosphoglycerate mutase | 1,339,734-1,341,302 | 2,3-bisphosphoglycerate-independent phosphoglycerate mutase  [EC 5.4.2.12] |
|  | Enolase | 1,214,473-1,215,198 | Phosphopyruvate hydratase [EC 4.2.1.11] |
|  | Pyruvate kinase | 2,613,075-2,614,832 | Pyruvate kinase [EC 2.7.1.40] |
| Gluconeogenesis only | PEP synthase | 2,587,877-2,590,234 | Phosphoenolpyruvate synthase [EC 2.7.9.2] |
|  | Fructose-1,6-bisphosphatase | 600,818-601,744 | Class I fructose-bisphosphatase [EC 3.1.3.11] |
|  | GAPDH type II | 1,352,371-1,353,438 | Type II glyceraldehyde-3-phosphate [EC 1.2.1.59] |
| Other | Fructokinase | 1,356,510-1,357,448 | Carbohydrate kinase, fructokinase [EC 2.7.1.4] |
|  | GFO domain | 1,190,747-1,191,880 | Gfo/Idh/MocA family oxidoreductase [EC 1.1.1.292] |
|  | XI/TIM domain | 1,191,909-1,192,880 | Sugar phosphate isomerase/epimerase, xylose isomerase  [EC 3.1.21.2] |

**Supplementary Fig. S1.** Time-course profile of dry cell weight (white bars) and total carotenoid production (open circles) under shaken flask cultivation at 37°C, 200 rpm.


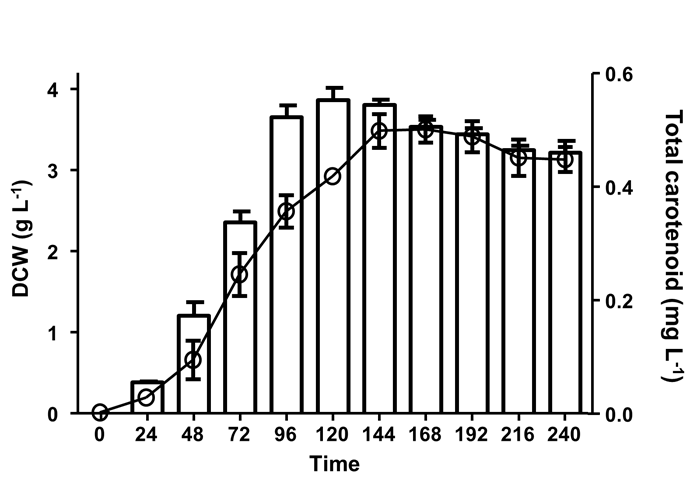


**Supplementary Fig. S2.** Pareto chart analysis: effect of the media component and other factors on total carotenoid production represented by t-value. The coded factors were X_1_ = sucrose, X_2_ = yeast extract, X_3_ = MgSO_4_·7H_2_O, X_4_ = pH, X_5_= incubation time, X_6_ = inoculum volume, X_7_ = KCl, X_8_ = CaCl_2_·6H_2_O, and X_9_ = NaCl.


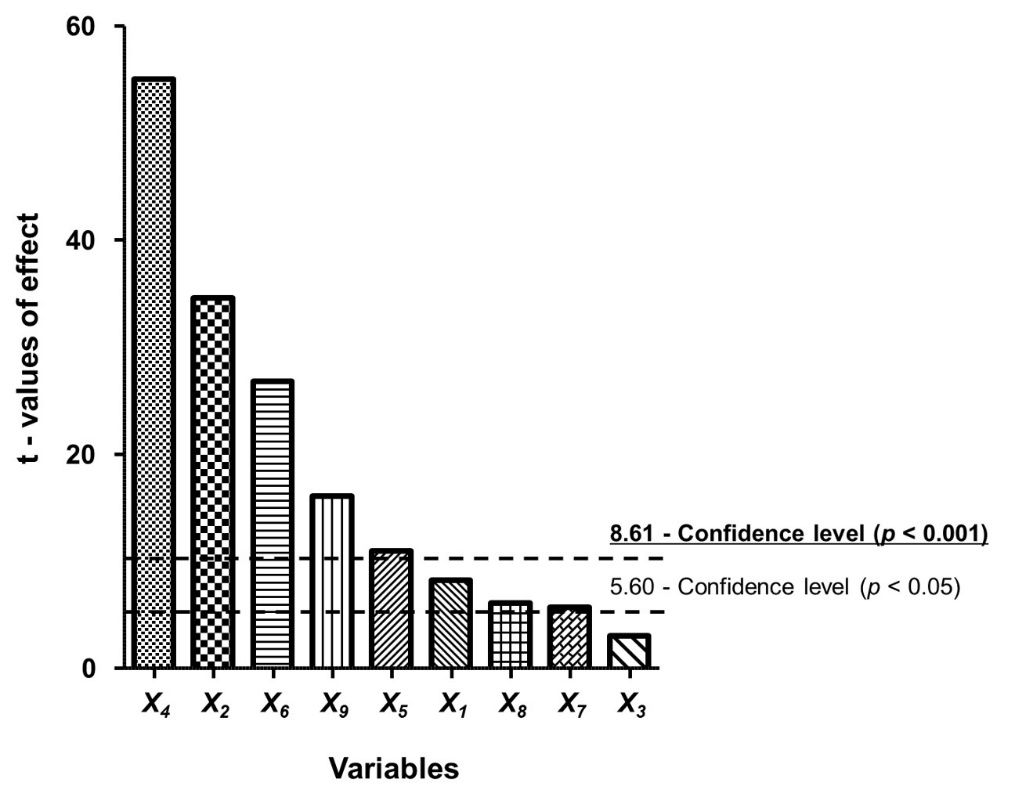


**Supplementary Fig. S3.** Time-course profiles of strain MBLA0099 on lab-scale 7 L fermenter showing 200 rpm agitation with (a) 0.5 vvm and (b) 1.0 vvm aeration with corresponding cell density (white bar), total carotenoid production (open circle), residual sucrose concentration (closed circle), dissolved oxygen (open square), and pH (closed triangle).


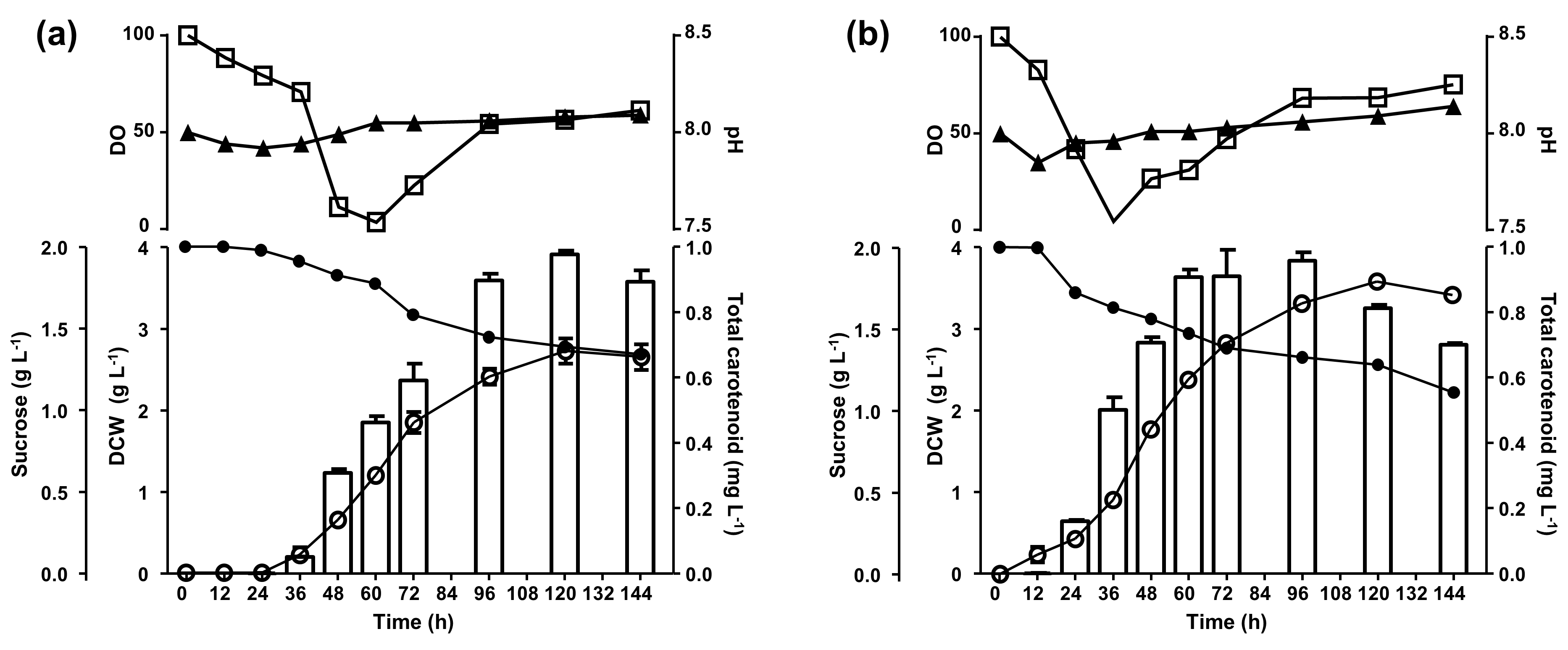


**Supplementary Fig. S4.** Time-course profiles of strain MBLA0099 on lab-scale 7 L fermenter showing 800 rpm agitation with (a) 0.5 vvm and (b) 1.0 vvm aeration with corresponding cell density (white bar), total carotenoid production (open circle), residual sucrose concentration (closed circle), dissolved oxygen (open square), and pH (closed triangle).


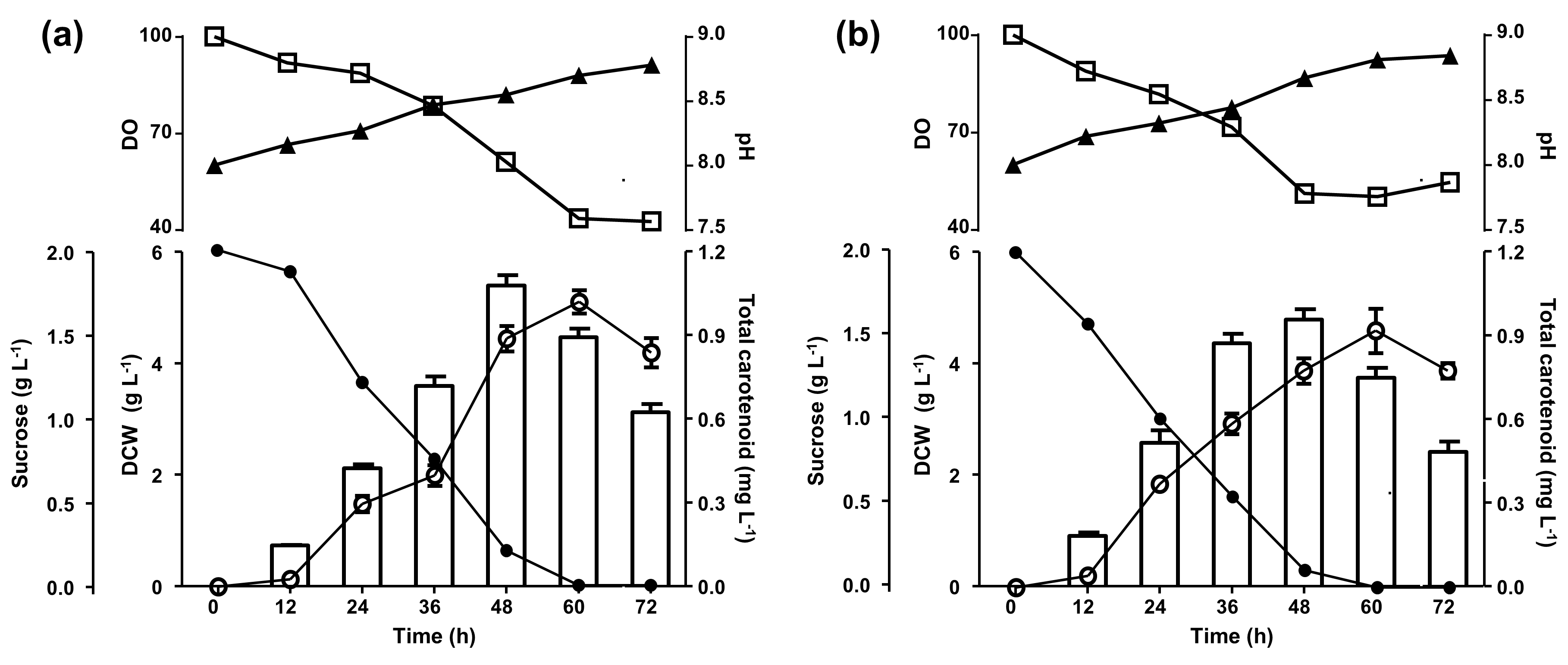


**Supplementary Fig. S5.** Cytotoxicity of BR extracts at different concentrations on Caco-2 cells. (a) Scheme of the cytotoxicity (b) cell viability by fluorescence detection and (c) cell morphology.


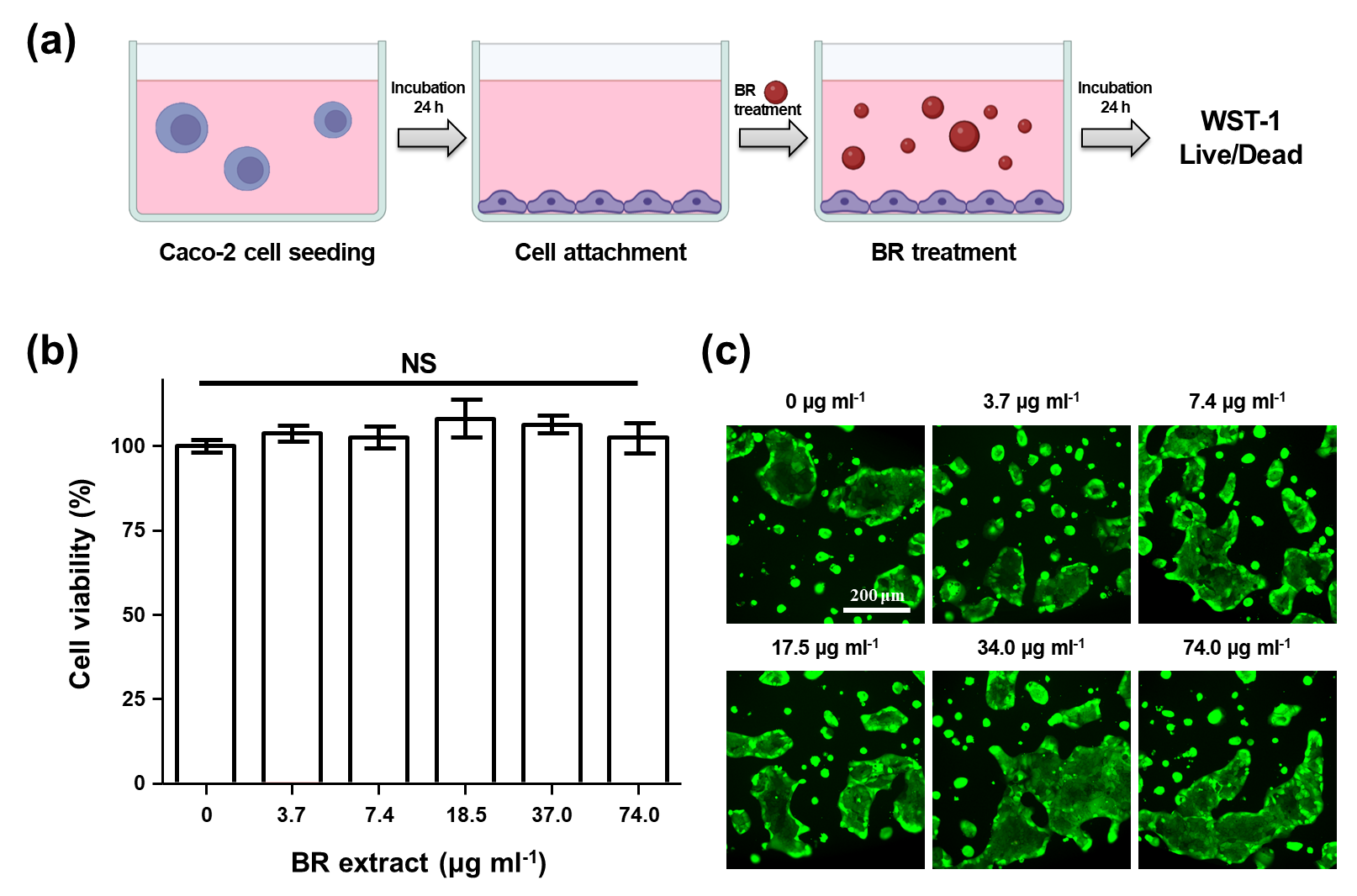

Supplement: Supplementary file 1 — Supplementary Material 1 [file 12934_2023_2274_MOESM1_ESM.docx]
